# Supplementary material for: Protein nanoparticle vaccines induce potent neutralizing antibody responses against MERS-CoV
Source: bioRxiv. 2024 Mar 14:2024.03.13.584735. Preprint. [Version 1] doi: 10.1101/2024.03.13.584735 (PMC10979991; doi:10.1101/2024.03.13.584735)
Supplement: Supplement 1 [file NIHPP2024.03.13.584735v1-supplement-1.pdf]

bioRxiv preprint doi: https://doi.org/10.1101/2024.03.13.584735; this version posted March 14, 2024. The copyright holder for this preprint (which was not certified by peer review) is the author/funder, who has granted bioRxiv a license to display the preprint in perpetuity. It is made available under aCC-BY 4.0 International license.

Supplemental Figures

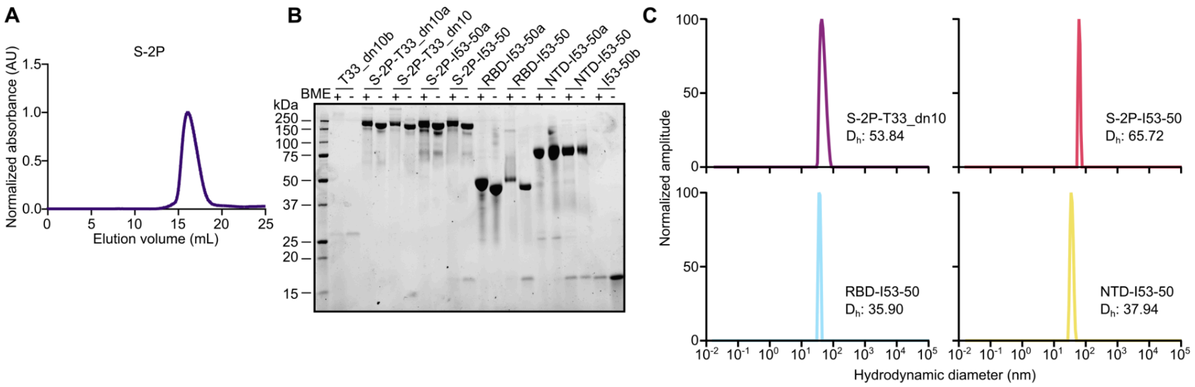

Fig. S1. Characterization of MERS immunogens.
(**A**) MERS-CoV S-2P SEC on a Superose 6 Increase 10/300 column. (**B**) SDS-PAGE of nanoparticle components before and after assembly under reducing and non-reducing conditions. BME, β-mercaptoethanol. (**C**) DLS of assembled nanoparticles measured on an UNcle (UNchained Labs). Hydrodynamic diameters (D_h) are indicated.

bioRxiv preprint doi: https://doi.org/10.1101/2024.03.13.584735; this version posted March 14, 2024. The copyright holder for this preprint (which was not certified by peer review) is the author/funder, who has granted bioRxiv a license to display the preprint in perpetuity. It is made available under aCC-BY 4.0 International license.

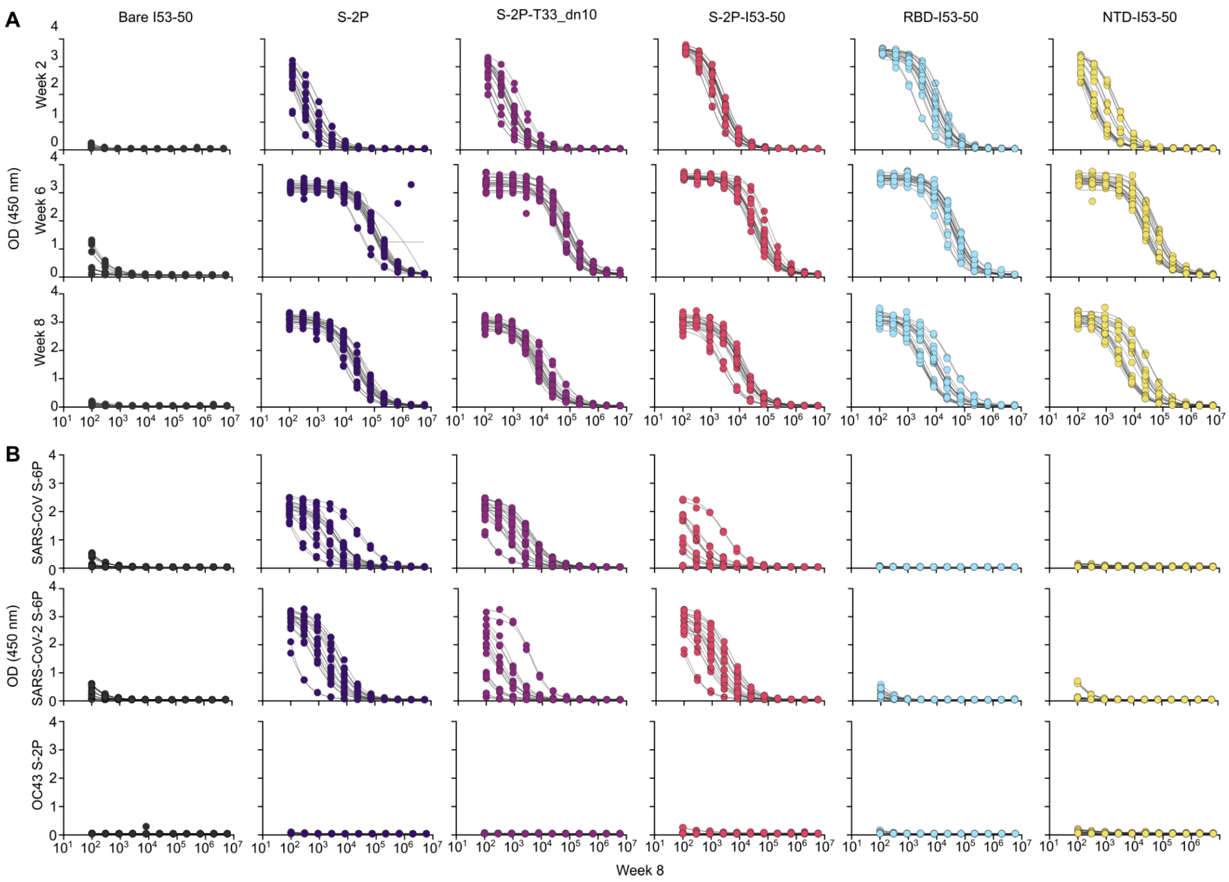

Fig. S2. Mouse serum binding to MERS-CoV S-2P, SARS-CoV-2 HexaPro S, and SARS-CoV HexaPro S.

(**A**) Raw week 2, 4, and 8 serum ELISA data against vaccine-matched (EMC) MERS-CoV S-2P. (**B**) Raw week 8 serum ELISA data against *top,* SARS-CoV HexaPro; *middle,* SARS-CoV-2 HexaPro; and *bottom,* OC43 S-2P.

bioRxiv preprint doi: https://doi.org/10.1101/2024.03.13.584735; this version posted March 14, 2024. The copyright holder for this preprint (which was not certified by peer review) is the author/funder, who has granted bioRxiv a license to display the preprint in perpetuity. It is made available under aCC-BY 4.0 International license.

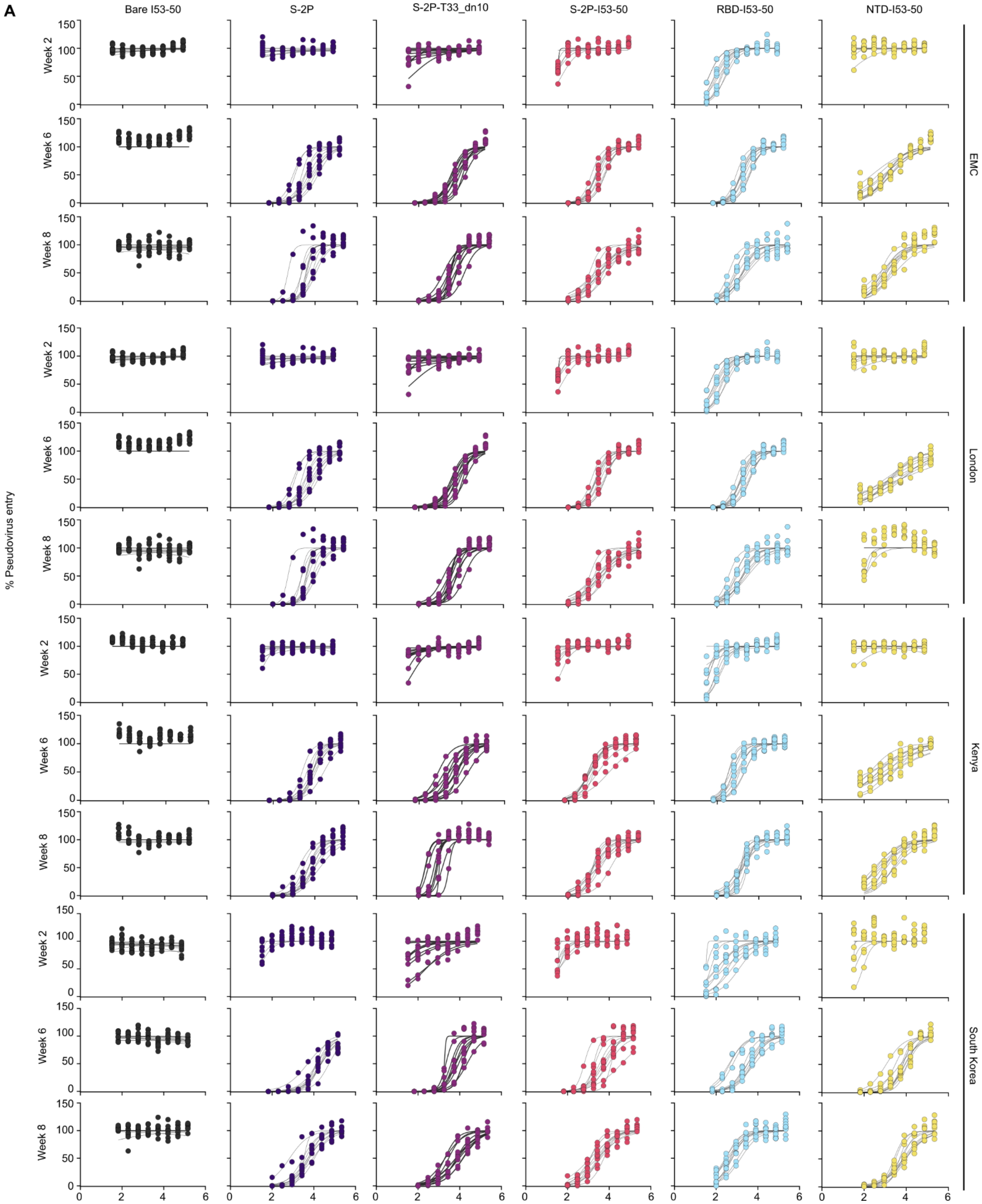

Fig. S3. Mouse serum pseudovirus neutralization.
(A) Raw neutralization data from weeks 2, 6, and 8 using pseudoviruses bearing the MERS-CoV EMC, London, Kenya, and South Korea variant spikes.

bioRxiv preprint doi: https://doi.org/10.1101/2024.03.13.584735; this version posted March 14, 2024. The copyright holder for this preprint (which was not certified by peer review) is the author/funder, who has granted bioRxiv a license to display the preprint in perpetuity. It is made available under aCC-BY 4.0 International license.

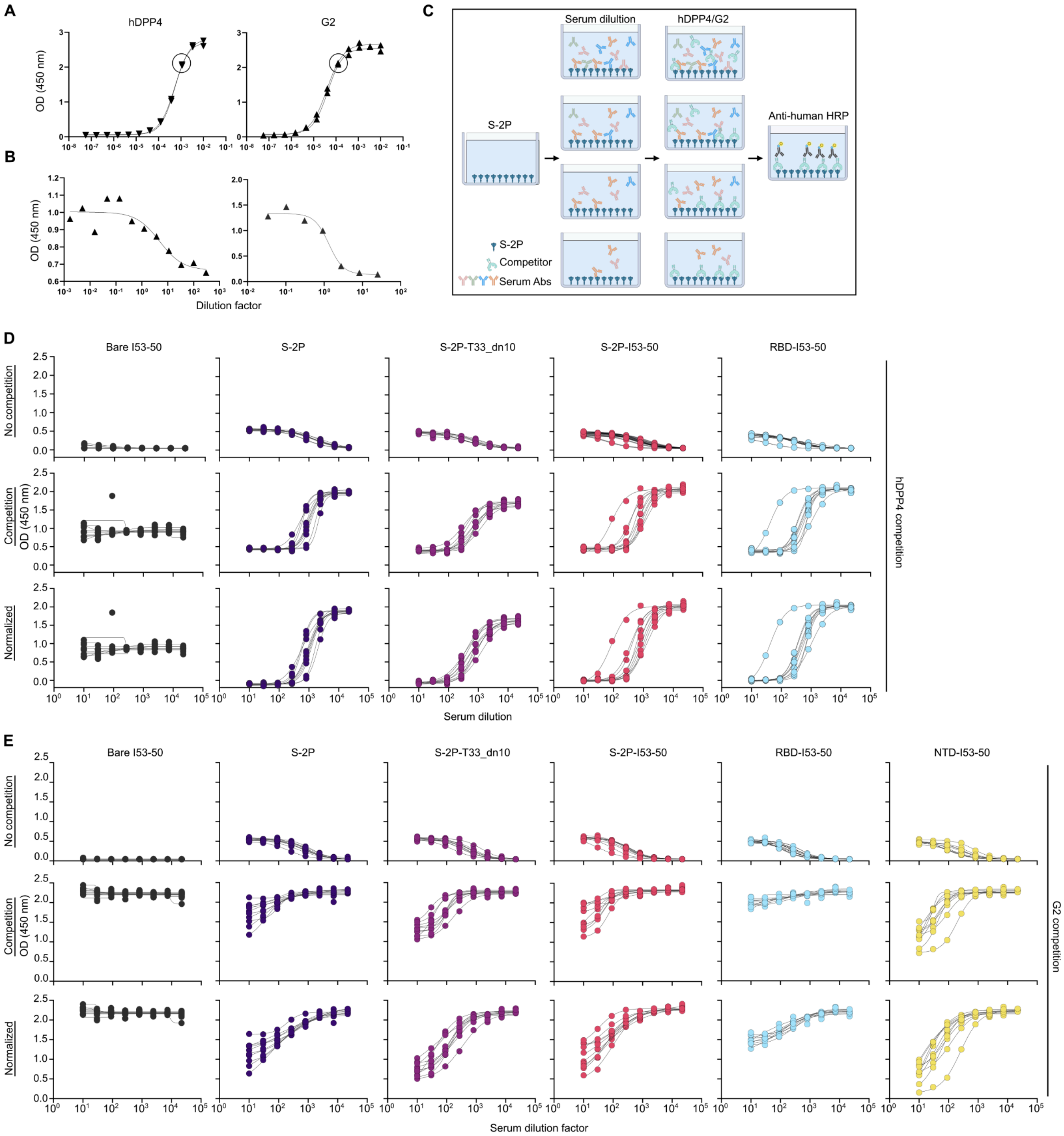

Fig. S4. Serum competition against hDPP4 and G2.
(**A**) Anti-S-2P ELISA with hDPP4-Fc and G2 to determine concentration of 80% maximum binding (circled). (**B**) Self competition with biotinylated hDPP4-Fc and G2 against S-2P. (**C**) Schematic of serum competition ELISA assay. Serum dilutions were incubated on plates coated with S-2P, followed by a fixed concentration of the competitor. Levels of bound competitors were measured as the final readout. (**D**) Per-mouse serum competition from week 8 against hDPP4-Fc binding to S-2P. Background serum reactivity to *top,* secondary antibody; *middle,* competition between hDPP4-Fc and serum; and *bottom,* normalized data obtained by subtracting background secondary antibody signal from competition. The arithmetic mean of the normalized data points at each dilution are shown in **Fig. 3A**. (**E**) Per-mouse serum competition from week 8 against G2 binding to S-2P, processed as in (D).

bioRxiv preprint doi: https://doi.org/10.1101/2024.03.13.584735; this version posted March 14, 2024. The copyright holder for this preprint (which was not certified by peer review) is the author/funder, who has granted bioRxiv a license to display the preprint in perpetuity. It is made available under aCC-BY 4.0 International license.

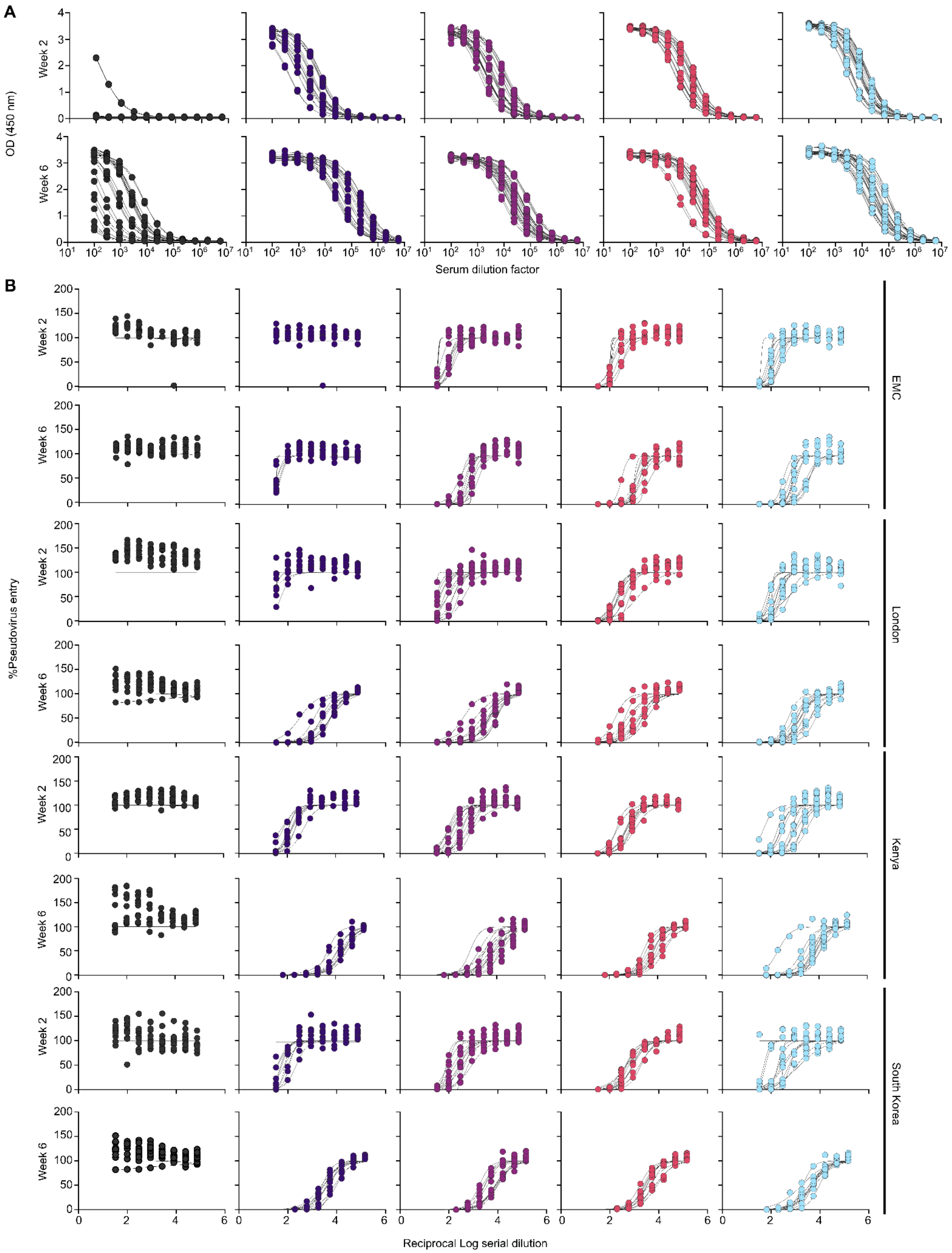

bioRxiv preprint doi: https://doi.org/10.1101/2024.03.13.584735; this version posted March 14, 2024. The copyright holder for this preprint (which was not certified by peer review) is the author/funder, who has granted bioRxiv a license to display the preprint in perpetuity. It is made available under aCC-BY 4.0 International license.

Fig. S5. 288/330$^{+/+}$ mouse serum binding to MERS-CoV S-2P and pseudovirus neutralization. (**A**) Raw week 2 and 6 serum ELISA data against vaccine-matched (EMC) MERS-CoV S-2P. (**B**) Raw neutralization data from weeks 2 and 6 using pseudoviruses bearing the MERS-CoV EMC, London, Kenya, and South Korea variant spikes.
